# Comprehensive Analysis of Differentially Expressed lncRNAs miRNAs and mRNA and Their ceRNA Network of Patients With Rare-Earth Pneumoconiosis

**DOI:** 10.3389/fgene.2021.700398

**Published:** 2021-07-19

**Authors:** Xue-min Shi, Yu-chao Bai, Yan-rong Gao, Ning Bu, Hai-yan Song, Li-hua Huang, Yu-hang Zhao, Su-hua Wang

**Affiliations:** School of Public Health, Baotou Medical College, Baotou, China

**Keywords:** long non-coding RNA, micro RNA, mRNA, competitive endogenous RNA, rare-earth pneumoconiosis

## Abstract

Rare-earth pneumoconiosis (REP) is the main occupational disease of rare earth exposed workers and there is no specific treatment. In this study, we performed high-throughput sequencing on the plasma of nine REP to describe and analyze the expression profiles of long non-coding RNA (lncRNA), micro RNA (miRNA) and mRNA and investigate their regulatory networks. Our results identified a total of 125 lncRNAs, 5 miRNAs, and 82 mRNAs were differentially expressed in the plasma of patients with REP. Furthermore, Ontology (GO) and Kyoto Encyclopedia of Genes and Genomes (KEGG) analysis were used to analyze the differentially expressed non-coding RNAs (ncRNA). We found the differential expression of ncRNA are mainly related to the response of cells to stimulation, Hedgehog signaling pathway and so on. We also constructed lncRNA-miRNA-mRNA networks to further explore their underlying mechanism and possible relationships in REP. We found that in the competitive endogenous RNA (ceRNA) networks, lncRNA acts as a sponge of miRNA to regulate the target gene. The expression results were verified by qRT-PCR and the protein interaction networks of differentially expressed genes were constructed via the STRING database. OncoLnc online platform was used to do the lung cancer survival analysis among the top five mRNA analyzed by Protein-protein interaction (PPI) network analysis. We found miR-16-2-3p may used as biomarker for REP, because it is closely related to the occurrence and prognosis of REP through inflammatory reaction and in lung squamous cell carcinoma, its expression levels were positively correlated with the overall survival rate of patients.

## Introduction

Rare earth (RE) recognized as “industry gold” is an indispensable strategic mineral resource in many fields, such as the national defense industry, new technology, and so on. China has unique rare earth resources. With the massive mining and processing of rare earths, the prevalence of rare-earth pneumoconiosis (REP) among workers exposed to rare earths is increasing year by year (Ji et al., 2017). REP is the main occupational disease of rare earth exposed workers and there is no effective treatment methods. Therefor it is necessary to further explore the unknown molecular mechanism and discover potential biomarkers.

With the development of biological sequencing technology, it has been gradually found that more than 70% of the genes in the human genome can be transcribed, and the transcripts contain non-coding RNA (ncRNA) (Jalali et al., 2016). Studies have shown that ncRNAs have regulatory significance in tumors (Evangelista et al., 2021; Viviana and Massimo, 2021). Experts have conducted extensive in-depth studies on pneumoconiosis, focusing on symptoms from oxidative stress to epithelial-mesenchymal transformation (EMT) and found ncRNAs are involved in the regulation of fibrosis process (Perdas et al., 2016; Sun et al., 2016). As one of ncRNA, previous studies have shown that miRNA is dysfunctional in cancer and is involved in a variety of cancer-related biological processes, including apoptosis, proliferation and EMT (Bartel, 2009; Wang et al., 2016). Because the expression of miRNA has obvious characteristics in different cancers, it has a great prospect in the clinical diagnosis of different diseases (Thum et al., 2008; Yamada et al., 2013; Mirzaei et al., 2016). Existing evidence shows that lncRNA can participate in multiple biological processes, including cell proliferation, cell cycle, cell differentiation and apoptosis (Zhang et al., 2019). Abnormal expression of lncRNAs has been associated with the occurrence and development of inflammation, cancer and immune diseases (Roux and Lindsay, 2015). But there are few researches on pathogenesis of REP at the genetic level.

In 2011, the competitive endogenous RNA (ceRNA) hypothesis was proposed (Leonardo et al., 2011). The ceRNA hypothesis details that all kinds of RNA molecules in the human body are closely connected and form a huge network of interactions. Under normal conditions, various types of molecules in the human body are in dynamic equilibrium. When the expression of one or more molecules is up-regulated or down-regulated, there will be a chain reaction that disrupts the homeostasis, and eventually leads to the occurrence of disease. A large number of experimental studies have confirmed lncRNA-miRNA-mRNA ceRNA regulatory networks in liver cancer, lung cancer, and breast cancer (He et al., 2020; Xiao et al., 2020; Luo et al., 2021). Study Yi et al. (2018) has found that lncRNA-ATB could adsorb miRNA200c and lead to the high expression of ZEB1 protein, which promoted the occurrence of pulmonary fibrosis induced by cellular EMT.

However, at present, the related research at gene level on REP has been rarely reported. Therefore, in this study, we analyses the expression profiles of ncRNA by RNA sequencing, and predicted related functions through Gene Ontology (GO) and Kyoto Encyclopedia of Genes and Genomes (KEGG), then established the ceRNA network and Survival Analysis to discover the relationships between ncRNAs and mRNA and do the lung cancer survival analysis. Our finding might discover new pathogenesis of SAP and provide new treatment ideas.

## Materials and Methods

### Research Object

Nine patients with REP diagnosed from a rare earth smelter from 2016 to 2018 were selected as the REP group. In addition, 9 persons who participated in the physical examination were selected as healthy controls. There was no smoking in REP group, and the age was 50.01 ± 4.16. In the healthy group, there was no smoking, no history of dust exposure, and no obvious abnormality was noted in DR chest X-rays. All subjects were male and there were no other detected pulmonary or bronchial diseases. The age and length of service of the two groups are compared. This study was subject to approval by the Medical Ethics Committee of Baotou Medical College and included the informed consent of all patients.

### Biological Sample Collection

The fasting venous blood (5 mL) was collected from individuals in EDTA anticoagulant collection vessels in the morning. After 1–2 h, plasma was centrifuged by 3,000 rpm and then packed into sterilized EP tubes. The isolated plasma was centrifuged for 10 min at 15,000 rpm to pellet cell fragments, and the plasma of each group was randomly mixed into three tubes to reduce individual differences. The plasma was stored on dry ice during transport and was brought back to the laboratory and stored at −80°C until testing.

### Extraction and Sequencing of RNA From Plasma

Total RNA from plasma was extracted using the TriZol (Invitrogen California, United States) reagent. The total RNA of the sample was detected by NanoDrop ND-2000 (Thermo Scientific Massachusetts, United States), and the integrity of RNA was detected by an Agilent Bioanalyzer 2100 (Agilent Technologies United States). The labeling efficiency of DNA probe and the result of chip hybridization is affected by the total RNA purity, so the extracted total RNA was purified by QIAGEN RNeasy Kit, and the purified total RNA was used for follow-up experiments. 250 ng of RNA was used for labeling and amplification. First-strand cDNA was synthesized from total RNA using the Promoter Primer and Affinity Script-RT kit. The second strand of cDNA was synthesized using an Anti-sense Promoter. cRNA was generated using T7 RNA polymerase. cRNA was labeled with the fluorescent dye Cyanine-3-CTP (Cy3) and then purified using the QIAGEN RNeasy Kit. Finally, the original image of product was scanned with an Agilent Scanner G5761A (Agilent Technologies, United States) after 17 h of rolling hybridization at 65 °C. Feature Extraction software (version12.0.3.1, Agilent Technologies, United States) was used for the extraction of the original image and the original data. Quantile standardization and subsequent processing were carried out using the GeneSpring software (version14.8, Agilent Technologies United States). The data were then filtered and at least one group of labeled probes were left behind for follow-up analysis in the case group and control group for comparison. A *T-*test was used to determine differential gene expression, and the screening criteria were a *P*-value ≤ 0.05 and difference multiple ≤ 2 for expression levels.

### Functional Enrichment Analysis

Gene Ontology (GO) enrichment analysis and Kyoto Encyclopedia of Genes and Genomes (KEGG) pathway analyses were used to clarify the unique biological significance of the differentially expressed genes found in the plasma samples of patients with REP. The function of differentially expressed RNA in REP was speculated by GO analysis, and genes were annotated and classified depending on molecular function, biological process, and cellular composition. *P* ≤ 0.05 considered that the difference was statistically significant. Pathway analysis can explain the biological function of these RNA genes in REP. Through the pathway analysis of differentially expressed RNA, we inferred relevant pathways and biological functions of differentially expressed genes. *P* ≤ 0.05 was used as the screening criterion for significant enrichment.

### Construction of the ceRNA Regulatory Network

In order to better understand the differential expression of RNAs in REP, we further constructed the lncRNA-miRNA-mRNA ceRNA regulatory network. We used miRanda and Target scan software to predict the target genes of differentially expressed miRNA and lncRNA, compared the target genes with the differentially expressed RNA we screened, excluded the target genes that were not in our screened RNA data set, constructed the lncRNA-miRNA-mRNA ceRNA regulatory network, and used Cytoscapesoftware to visualize the data.

### Real-Time PCR Validation of Differentially Expressed RNAs

A total of 14 research objects, including 7 patients with REP and 7 healthy persons were used for validation. Total RNA was extracted by the miRcute miRNA Extraction and Separation kit. miRNA cDNA first chain synthesis and quantitative fluorescence detection were performed using the miRcute Enhanced miRNA cDNA First Chain Synthesis Kit and miRcute Enhanced miRNA Fluorescence Quantitative Detection kit. For lncRNA, mRNA cDNA first chain synthesis and fluorescence quantitative detection using lnRcute lncRNA cDNA first chain synthesis kit and lnRcute lncRNA fluorescence quantitative detection kit. All primer sequences are shown in the table below.

### Protein-Protein Interaction (PPI) Network Construction

The Retrieval of Interacting Genes (STRING) database tool (string-db.org) was utilized to figure out the interactive relationships of differentially expressed mRNA. Interacting pairs with a confidence score greater than 0.4 were considered as significant and retained. Built on our STRING results, the first five genes with the highest PPI score were screened out for further survival analysis. The PPI network was visualized utilizing Cytoscape software.

### Survival Analysis

The OncoLnc network platform collected the survival data of 21 kinds of tumors in TCGA, from a total of 8,647 patients, as well as the corresponding mRNA and miRNA expression profile data. At the same time, the lncRNA expression data from the MiTranscriptome project, which can easily mine the survival-related genes in all kinds of tumors, were collected. We used this platform to analyze the survival of mRNA, which ranked among the top five in PPI network analysis, differentially expressed miRNA and lncRNA in lung cancer. The patients were subdivided into a high-expression group and a low-expression group according to the average expression of the gene as the median. *P* < 0.05 indicated that there was a significant difference between the two groups.

### Statistical Analysis

All data are shown as mean ± SD. The fold change value and *P*-value were used to evaluate the differences of lncRNA, miRNA, and mRNA expression between the REP and healthy group. *P* < 0.05 was considered to be statistically significant.

## Results

### Differential Expression of lncRNAs, miRNAs, and mRNAs

**FIGURE 1:**
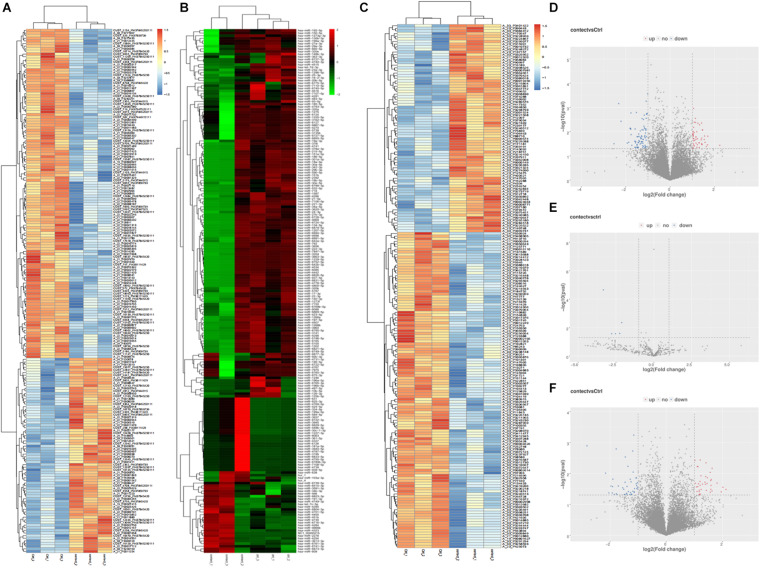
Differentially expressed lncRNAs, miRNAs, mRNAs heat map, volcano map representing the differentially expressed genes from the plasma of patients with rare-earth pneumoconiosis. **(A)** Differential expression of the lncRNA heat map. **(B)** Differential expression of the miRNA heat map. **(C)** Differential expression of the mRNA heat map. **(D)** Differentially expressed lncRNA volcano map. **(E)** Differentially expressed miRNA volcano map. **(F)** Differentially expressed mRNA volcano map.

Compared to the control group, there were 125 lncRNAs, 5 miRNAs, and 82 mRNAs that were differentially expressed in the plasma of REP patients. The heat map and volcanic map depicting these data are shown in Figure 1. Among them, 49 lncRNAs were up-regulated and 76 were down-regulated, 38 mRNAs were up-regulated and 44 were down-regulated, and all the differentially expressed miRNAs were down-regulated. The differentially expressed RNAs are shown in Table 1.

**TABLE 1:** Differentially expressed lncRNAs, mi RNA and mRNA in the plasma of REP patients.

**Differentially expressed lncRNAs**				**Differentially expressed miRNAs**				**Differentially expressed mRNAs**			
**Gene symbol**	**log2 fold change**	**Regulation**	**pval**	**Gene symbol**	**log2 fold change**	**Regulation**	**pval**	**Gene symbol**	**log2 Fold Change**	**Regulation**	**pval**
lnc-ELAC1-2	2.24	Up	0.04	STK10	2.12	Up	0.03	hsa-miR-4515	−3.33	Down	0.00
LINC02699	1.70	Up	0.02	CLCNKA	2.01	Up	0.03	hsa-miR-1296-5p	−2.79	Down	0.03
lnc-PARD3B-2	1.68	Up	0.03	CYP2D8P	1.95	Up	0.04	hsa-miR-7106-5p	−2.53	Down	0.03
CEP83-AS1	1.63	Up	0.04	ZNF614	1.81	Up	0.02	hsa-miR-16-2-3p	−2.26	Down	0.03
RP11-53B2.1	1.57	Up	0.03	LOC100128751	1.63	Up	0.04	hsa-let-7d-3p	−2.12	Down	0.00
SRD5A3-AS1	1.55	Up	0.02	LINC02864	1.60	Up	0.02				
AC131009.1	1.53	Up	0.02	FAM27B	1.59	Up	0.02				
TUNAR	1.46	Up	0.01	TIGD7	1.58	Up	0.03				
LINC00968	1.45	Up	0.01	ANKRD62	1.58	Up	0.02				
lnc-NDUFB5-1	1.44	Up	0.03	KRR1	1.56	Up	0.04				
linc-GLIS2-1	−2.53	Down	0.03	SLC22A18AS	−2.82	Down	0.00				
lnc-VIM-1	−2.32	Down	0.00	B3GNT3	−2.16	Down	0.01				
LOC101929666	−2.21	Down	0.00	ITGA6	−2.04	Down	0.04				
lnc-STX18-2	−1.93	Down	0.03	KDM5C	−1.97	Down	0.03				
lnc-REG1B-2	−1.87	Down	0.03	EFCAB11	−1.92	Down	0.04				
lnc-REG1B-4	−1.81	Down	0.01	TMEM237	−1.91	Down	0.03				
AP005717.1	−1.79	Down	0.01	H2AFV	−1.90	Down	0.02				
lnc-MFSD9-4	−1.77	Down	0.01	KRTAP27-1	−1.85	Down	0.05				
AC024560.1	−1.77	Down	0.00	CD86	−1.78	Down	0.00				

### GO and KEGG Analyses of Differentially Expressed lncRNAs, miRNAs, and mRNAs

**FIGURE 2:**
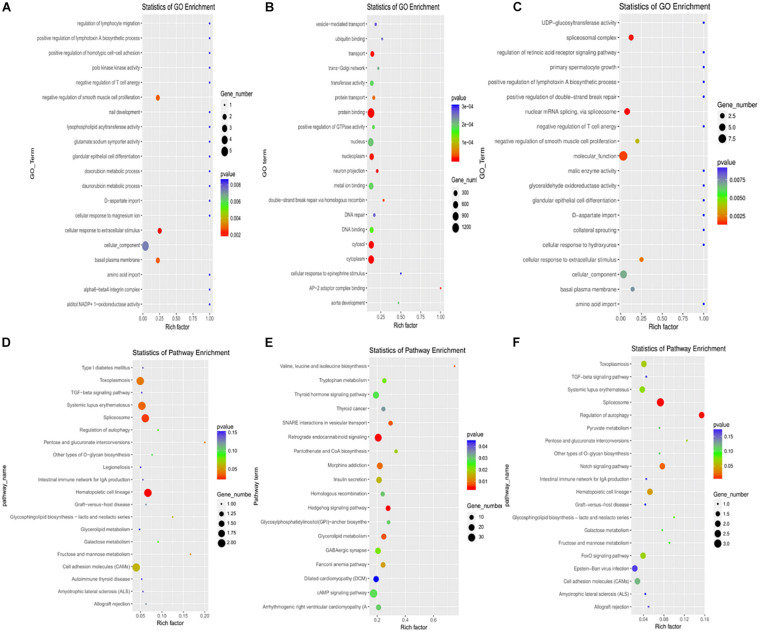
Differentially expressed lncRNAs, miRNAs, and mRNAs GO enrichment scatter plots and KEGG enrichment scatter plots in plasma of rare-earth pneumoconiosis patients. **(A)** Differentially expressed lncRNA GO enrichment. **(B)** Differentially expressed miRNA GO enrichment. **(C)** Differentially expressed mRNA GO enrichment. **(D)** Differentially expressed lncRNA KEGG enrichment. **(E)** Differentially expressed miRNA KEGG enrichment. **(F)** Differentially expressed mRNA KEGG enrichment.

According to GO analysis, the main enriched functions of differentially expressed lncRNAs were involved in the negative regulation of smooth muscle cell proliferation, cell response to extracellular stimulation, Polo-like kinase activity, and nuclear mRNA splicing. The main enrichment functions of differentially expressed miRNAs were the regulation of GTP enzyme activity, DNA repair, ion migration, and double-strand break repair by homologous recombination. The main enriched functions of differentially expressed mRNAs were involved in transmembrane signal transduction receptor activity, autophagy vacuole formation, splicing of nuclear mRNA through splice bodies, and cell response to extracellular stimulation. The GO enrichment scatter diagram is shown in Figure 2.

By KEGG analysis, the differentially expressed lncRNAs were mainly enriched in fructose and mannose metabolism, pentose and glucuronide transformation, and splicing. The differentially expressed miRNAs were mainly enriched for the Hedgehog signaling pathway, cAMP signal pathway, glycerol metabolism, pantothenic acid, and CoA biosynthesis. Finally, the differentially expressed mRNAs were mainly enriched in autophagy regulation, Notch signal pathway, and hematopoietic cell pedigree. The KEGG enrichment scatterplot is shown in Figure 2.

### Construction of a lncRNA-miRNA-mRNA ceRNA Regulatory Network

**FIGURE 3:**
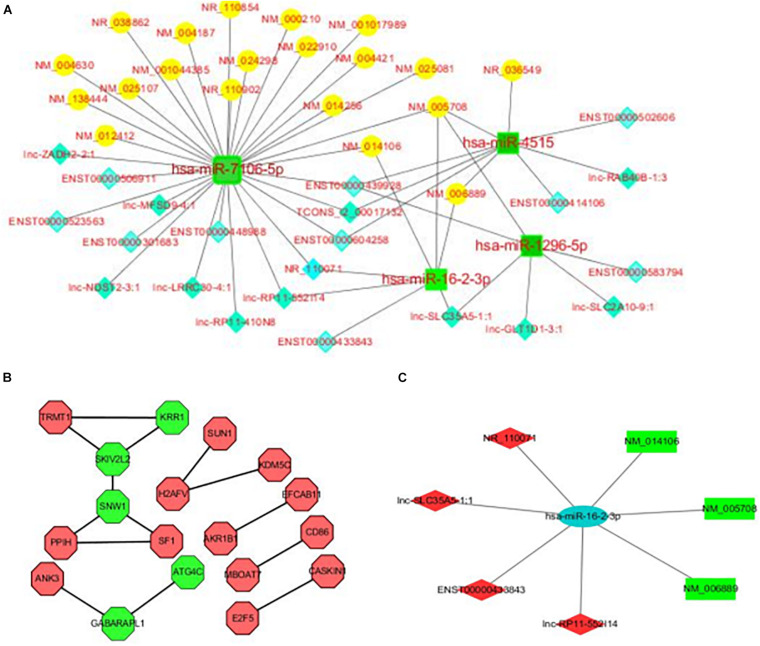
**(A)** LncRNA-miRNA-mRNA ceRNA regulatory network in the plasma of rare-earth pneumoconiosis patients. The blue diamond is lncRNA, the green square is miRNA, and the yellow circle is mRNA. **(B)** Differentially expressed mRNA PPI network in rare-earth pneumoconiosis patients. Green hexagons are the top 5 genes. **(C)** Hsa-miR-16-2-3p related ceRNA regulatory network in the plasma of rare earth pneumoconiosis patients. The blue ellipse is miRNA, the green rectangle is mRNA, and the red quadrilateral is lncRNA.

In order to better understand the role and interactions of differentially expressed lncRNA, miRNA, and mRNA in REP, we further constructed a lncRNA-miRNA-mRNA-related ceRNA regulatory network of REP, as shown in Figure 3A. The network involves 21 lncRNAs, 4 miRNAs, and 21 mRNAs. Each differentially expressed gene can be associated with one or more miRNAs. For example, The network has multiple common nodes, such as lnc-SLC35A5-1:1 interact with hsa-miR-16-2-3p and hsa-miR-4515, and these two miRNA both interact with NM_005708. Figure 3C is the hsa-miR-16-2-3p ceRNA network, which shows hsa-miR-16-2-3p has been connected with 4 lncRNAs, including NR_110071, lnc-SLC35A5-1:1, ENST00000433843 and lnc-RP11-552114, 3 mRNA, including NM_014106, NM_005708, and NM_006889.

### Verification of Differential Expression of lncRNAs, miRNAs, and mRNAs

**FIGURE 4:**
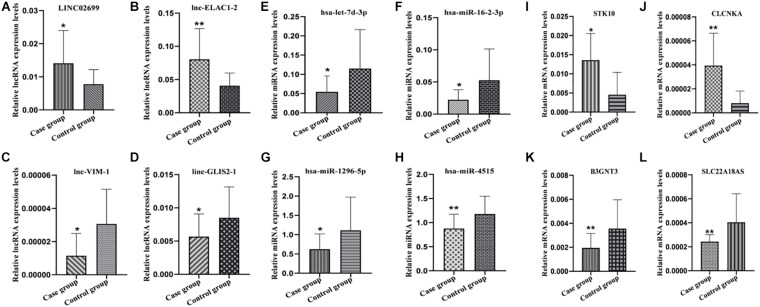
Partial RNA qRT-PCR verification results. **(A–D)** lncRNA qRT-PCR results. **(E–H)** miRNA qRT-PCR results. **(I–L)** mRNA qRT-PCR results. *P ≤ 0.05, **P ≤ 0.01.

In order to verify the reliability of the results of our high-throughput RNA sequencing, four items (*p* < 0.05, Fold change ≥ 2) were selected from the differential expression lncRNAs, miRNAs and mRNAs for qRT-PCR verification. The lncRNA, miRNA, and mRNA verification results are shown in Figure 4, respectively. Our verification results were consistent with the sequencing results, so these all proved the accuracy of the microarray results.

### Construction of a Differentially Expressed mRNA PPI Network

Functional links between proteins can be inferred from the genomic correlation between the genes that encode them. In order to study the interaction between differentially expressed proteins in REP, a differentially expressed mRNA PPI network map was further constructed, as shown in Figure 3B. The network consists of 18 nodes and 14 sides. The top five genes in the PPI relationship score were SNW family gene 1 (SNW1), viral activity killer (SKIV2L2), small subunit component homolog (KRR1), γ-aminobutyric acid receptor-related protein 1 (GABARAPL1), and cysteine protease (ATG4C).

### Survival Analysis of Select Differentially Expressed lncRNAs, miRNAs, and mRNAs

**FIGURE 5:**
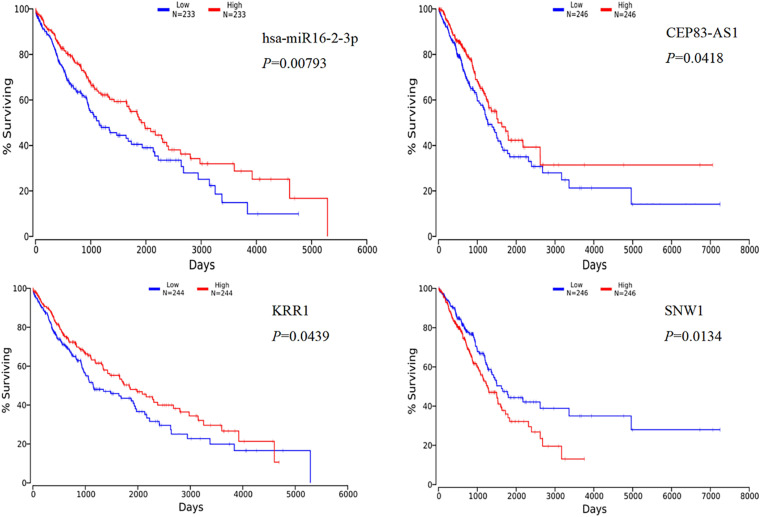
Kaplan-Meier curve analysis of miRNA, lncRNA, mRNA and overall survival in rare-earth pneumoconiosis patients.

Through the online platform OncoLnc, we performed survival analysis of the top five differentially expressed mRNAs that were obtained by PPI network analysis, and the differentially expressed miRNAs and lncRNAs in lung cancer, including lung squamous cell carcinoma (LUSC) and lung adenocarcinoma (LUDA) were studied. The results of the Kaplan-Meier curve analysis showed that the expression levels of hsa-miR-16-2-3p and KRR1 were positively correlated with the overall survival rate of LUSC, *P* < 0.05, the expression level of CEP83-AS1 was positively correlated with the overall survival rate in LUDA, and the expression level of SNW1 was negatively correlated with the overall survival rate, *P* < 0.05 (Figure 5).

## Discussion

At present, more and more studies have shown that ncRNAs play an important role in the occurrence and development of various diseases. Our research studied the lncRNA miRNA and mRNA expression profile REP plasma to explore the underlying function of lncRNA and miRNA. In total, we identified 125 differentially expressed lncRNA, 49 of which were up-regulated and 76 of which were down-regulated, 5 differentially expressed miRNAs and 82 differentially expressed mRNA, 38 were up-regulated and 44 were down-regulated. Among them, the first two up-regulated and down-regulated RNA were verified by qRT-PCR, and the results were consistent with the sequencing results.

In the screening of miRNA expression profile of REP patients, there were 5 differentially expressed miRNA, and all of them were down-regulated, namely hsa-let-7d-3p, hsa-miR-16-2-3p, hsa-miR-1296-5p, hsa-miR-7106-5p, and hsa-miR-4515. Let-7d is one of the earliest and most deeply studied miRNA. Researches have shown that the expression of let-7 is decreased in a variety of tumor cells (Perdas et al., 2016; Sun et al., 2016), while restoring the expression of let-7 in tumor cells can inhibit the migration and invasion of tumor cells, so the let-7 family often plays the role of tumor suppressor genes. Recent studies have shown that let-7 plays an important role in fibrotic diseases (Pandit et al., 2010; Wang et al., 2016; Chen et al., 2017; Matsuura et al., 2018), as an important member of the let-7 family, let-7d has the same function. For example in the process of pulmonary fibrosis, transforming growth factor β1 (TGF-β1) can bind to the let-7d promoter through Smad3, resulting in down-regulation of let-7d, which further up-regulates the high mobility group protein A2 (HMGA2) of its target gene, promotes EMT, and leads to fibrosis (Pandit et al., 2010). There is also a low expression of let-7d in the EMT process of renal fibrosis, while the high expression of let-7d can inhibit the EMT process of adrenocortical cells induced by TGF-β1 (Wang et al., 2016). Our results are consistent with the above researches, let-7d expression of REP shows down-regulated.

Beside let-7d, the other four miRNA are rarely reported in pneumoconiosis. But for miR-16-2-3p, its role in other diseases has been reported such as in ovarian cancer. Study has found that miR-16-2-3p might be involved in the progression of Ig A nephropathy (Yan et al., 2015) and non-syndromic cleft lip (Zou et al., 2016). In a study by Liang et al. (2015), it was found that miR-16-2-3p negatively regulates the protein expression level of IL-6 by binding to the 3′UTR of IL-6 for post-transcriptional regulation. As we all know, IL-6, as a pro-inflammatory factor, plays an important role in the occurrence and development of pneumoconiosis. Our previous study have shown that inflammatory factors in the blood of workers exposed to rare earths are increased.

Studies have found that lncRNAs which have miRNA response elements have the ability of sponge miRNA (Arun et al., 2018). So LncRNAs can affect the abundance of target gene mRNA and thus its protein level through sponge miRNA. LncRNAs are involved in the occurrence and development of many diseases by acting as a competitive endogenous RNA (ceRNA), such as tumors (Chao et al., 2020), non-small cell lung cancer (Wang et al., 2020), nervous system development (Yang et al., 2018), metabolic diseases (Zhu et al., 2019), cardiovascular diseases (Cai et al., 2020). In order to better understand the role and interactions of differentially expressed lncRNA, miRNA, and mRNA in REP, we constructed a lncRNA-miRNA-mRNA-related ceRNA regulatory network. And the ceRNA network showed that the expression of lncRNAs and mRNAs are consistent in trend, and the main trend is up-regulated. Some researchers also found that the expression trends of lncRNAs and mRNAs are consistent when they analyzing co-expression of lncRNAs-mRNAs in other disease (Feng et al., 2018; Wang et al., 2021). This result proves that some lncRNAs act as miRNA sponge to affect one or more downstream gene expression and participate in the occurrence and development of REP. Then, we focused on miR-16-2-3p and its upstream and downstream RNA and constructed miR-16-2-3p ceRNA regulatory network. We found that miR-16-2-3p can affect the NM_006889 which is downstream gene of CD86. CD86 is a prominent surface molecule of dendritic cells and plays an important role in the immune system. Another study found that the increase of IL-6 can promote the expression of CD86 and enhance its ability to stimulate the proliferation of allogeneic T cells. Meanwhile, miR-16-2-3p also connected with NM_006889, which is related to lncRNA small nucleolar RNA host gene 5 (SNHG5). SNHG5 has been shown to be involved in the development and tumorigenesis of a variety of cancers (liver cancer, breast cancer, osteosarcoma, colorectal cancer; Ju et al., 2018; Li et al., 2018; Chi et al., 2019; Zhang et al., 2019; Li et al., 2020). Its disorder is closely related to metastasis, pathological staging, and prognosis (Li et al., 2020; Shen et al., 2020). For example, SNHG5 is also involved in the p38/MAPK signal pathway, Wnt/CTNNB1, and other pulmonary fibrosis-related signaling pathways (Chen et al., 2019; Hu et al., 2019). So it may be used as a key RNA for further study of. This result proves that miR-16-2-3p may be involved in the key process of immune response and pulmonary fibrosis-related signaling pathways. MiR-16-2-3p and SNHG5may be as a potential specific biomarker of the pathogenesis of REP.

Measnwhile the GO enrichment analysis results indicated that these differentially expressed miRNA were participated in the response of cells to extracellular stimulation, cell response to DNA damage stimulation, cell adhesion. KEGG analysis showed that the differential miRNA was mainly concentrated in the Hedgehog signaling pathway, pantothenic acid and CoA biosynthesis. Among these, Hedgehog signaling pathway is activated in different tumors, which may promote the occurrence of multiple types of tumors by promoting the process of tumorigenesis and metastasis (Bailey et al., 2007; Aval et al., 2017). These results suggesting differentially expressed miRNA may related to the process of EMT, inflammatory reaction and Hedgehog signaling pathway.

In addition, in order to better understand the relationship between the differentially expressed mRNAs, we used the STRING database to analyze the differentially expressed mRNAs and construct a PPI network graph, which consists of 18 nodes and 14 edges. Among them, SNW1 had the highest score of PPI relationship, which can fully activate the TGF-β/Smad3 signaling pathway by interacting with Smad3. At the same time, it was also found to be involved in NF-κB and Notch signaling pathways, which are closely related to the occurrence and development of pneumoconiosis (Verma et al., 2019). ATG4C is a molecule related to autophagy. Other study Gao et al. (2016) found that ATG4c is targeted by miR-142-3p and inhibits its expression in mouse macrophages, resulting in a significant down-regulation of LC3 II protein, thus regulating autophagy. The above two molecules can be used as key targets for further study of the mechanism of REP.

At last, we explore the survival analysis of the differentially expressed lncRNA, miRNA, and mRNA with the top five scores obtained by PPI network analysis in patients with REP. We found that four RNAs were significant in the survival analysis of lung cancer, in which the expression levels of miR-16-2-3p, lnc CEP83-AS1, and KRR1 were positively correlated with the overall survival rate, while the expression levels of SNW1 were negatively correlated with the overall patient survival rate. Study Maximov et al. (2019) found that miR-16-2-3p plays an important role as a tumor suppressor gene in osteosarcoma. In this analysis, miR-16-2-3p also showed its anti-cancer effect, which was positively correlated with the overall survival rate of patients with lung cancer, but it was down-regulated in patients with REP. Whether the down-regulation of miR-16-2-3p will increase the risk of lung cancer in patients with REP remains to be further studied.

## Conclusion

In conclusion, we found 125 lncRNA, 5 miRNA, and 82 mRNA differentially expressed in the plasma of patients with REP and constructed a REP-related ceRNA network. In particular, MiR-16-2-3p and SNHG5 is closely related to the occurrence and prognosis of REP through inflammatory reaction, which could be as biomarker for REP.

## Data Availability Statement

The datasets generated for this study can be provided if requested. Requests to access these datasets should be directed to the corresponding authors.

## Ethics Statement

The studies involving human participants were reviewed and approved by the Medical Ethics Committee of Baotou Medical College. The patients/participants provided their written informed consent to participate in this study.

## Author Contributions

S-hW, X-mS, and Y-hZ designed the research. Y-cB, Y-rG, and L-hH carried out the data collection and data analysis. NB and H-yS collected clinical samples. X-mS and Y-cB wrote the manuscript. All authors read and approved the final manuscript.

## Conflict of Interest

The authors declare that the research was conducted in the absence of any commercial or financial relationships that could be construed as a potential conflict of interest.
